# Utility of combining spectral domain optical coherence tomography structural parameters for the diagnosis of early Glaucoma: a mini-review

**DOI:** 10.1186/s40662-018-0101-6

**Published:** 2018-04-15

**Authors:** Jean-Claude Mwanza, Joshua L. Warren, Donald L. Budenz

**Affiliations:** 1 Department of Ophthalmology, School of Medicine, University of North Carolina at Chapel Hill, Chapel Hill, NC USA; 2 Department of Biostatistics, Yale University, New Haven, CT USA

**Keywords:** Optical coherence tomography, Combination of parameters, Early glaucoma

## Abstract

Optical coherence tomography (OCT) has moved to the forefront of imaging modalities in the management of glaucoma and retinal diseases. It is modifying how glaucoma and glaucoma progression are diagnosed clinically and augmenting our understanding of the disease. OCT provides multiple parameters from various anatomic areas for glaucoma diagnosis, evaluation of treatment efficacy, and progression monitoring. While the use of multiple parameters has increased the likelihood of detecting early structural changes, diagnosing glaucoma in early stages is often challenging when the damages are subtle and not apparent on OCT scans, in addition to the fact that assessment of OCT parameters often yields conflicting findings. One promising approach is to combine multiple individual parameters into a composite parameter from the same test to improve diagnostic accuracy, sensitivity, and specificity. This review presents current evidence regarding the value of spectral domain OCT composite parameters in diagnosing early glaucoma.

## Background

Glaucoma is a slowly progressive degenerative optic neuropathy characterized by the death of retinal ganglion cells (RGCs) and their axons, with associated morphologic changes to the optic nerve head (ONH), retinal nerve fiber layer (RNFL), and ganglion cell-inner plexiform layer (GCIPL). In most cases, the disease slowly leads to complete blindness if inadequately or not treated. Both medical and surgical treatment are often able to significantly slow the disease progression, which demonstrates the critical importance of accurate and early detection of the disease for early initiation of treatment [1]. Over the years, a number of imaging devices (i.e., retinal thickness analyzer, scanning laser polarimetry, and confocal scanning laser ophthalmoscopy) have been developed and used in the clinical setting to aid the clinician in the diagnosis and monitoring of glaucoma [2]. These modalities have since been supplanted by optical coherence tomography (OCT), particularly its spectral domain variant.

OCT has rapidly become the most widely used imaging modality for glaucoma management. Since its commercialization, it has revolutionized the management of retinal diseases (i.e. AMD, diabetic maculopathy, macular hole, central serous chorioretinopathy, retinal vein occlusions, and vitreo-retinal interface disorders) and glaucoma. In glaucoma, OCT provides objective, precise, and highly reproducible quantitative evaluation of inner retinal layers and the ONH [3–9]. Since diagnosing glaucoma is often unequivocal in moderate to advanced stages, imaging of the ONH, RNFL, and macula is, therefore, more valuable in the diagnosis of early than moderate to advanced disease. This review presents a compilation of available data on the usefulness of spectral domain OCT (SDOCT) in diagnosing early glaucoma by combining its parameters.

## Diagnosis of Glaucoma

While identification of glaucomatous optic neuropathy in moderate to advanced cases is often apparent clinically, diagnosing glaucoma in early stages can be challenging. Reasons for the challenge include the symptomless nature of the disease until a substantial number of RGCs and axons have been lost, the fact that no damage can be detected at the stage of RGC apoptosis along the glaucoma continuum [10], the wide interindividual variation in the anatomy of the ONH and RNFL, and the lack of a gold standard for establishing the diagnosis. Studies have shown that glaucomatous structural changes often precede functional loss [11–13], implying that in some patients with early stage (i.e., pre-perimetric glaucoma) effort should be made to establish the diagnosis based on structural changes alone. This is crucial because waiting for more visible signs of the disease would correspond to allowing the occurrence of some irreversible damage. Thus, identification of early damage to ocular structures affected by the disease is of paramount importance for early treatment to prevent irreversible functional loss.

SDOCT glaucoma modules currently include single parameters from the ONH, peripapillary RNFL, and macular GCIPL and/or ganglion cell complex (GCC). One device, Spectralis OCT by Heidelberg, also provides total retina thickness measurements in the macula. There are currently more than a dozen OCT parameters for glaucoma assessment; this number varies slightly between platforms. A number of other parameters have been described (i.e., lamina cribrosa depth or LCD [14], lamina cribrosa curvature index or LCCI [15], neuroretinal rim minimum distance band or MDB [16]) that are not currently reported on OCT printouts. Despite good diagnostic abilities of single parameters in early glaucoma, SDOCT devices still falsely classify healthy eyes as having glaucoma or miss the diagnosis of early glaucoma in substantial proportions of subjects [17–22]. Use of more than one individual parameter from the ONH, RNFL, and GCIPL or GCC for glaucoma assessment is therefore justified because it increases the likelihood of detecting a structural abnormality in at least one anatomic area. Indeed, findings from the three areas do not always show agreement. The caveat of such an approach is that it may increase the rate of false-positive conclusions unless appropriate corrections for multiple comparisons are made.

## Combination of parameters

There is abundant and convincing in vivo evidence of the association between glaucoma and structural damage to the ONH, RNFL, and macular GCIPL or GCC. OCT provides proof regarding qualitative and quantitative information collected on multiple parameters. The diagnosis is then based on careful interpretation of data on parameters from these anatomic structures combined with the clinical impression from the visual field and ocular examination. While the ideal situation to ascertain the diagnosis is to have an agreement among results on parameters from anatomic areas, that is not always the case in reality. Indeed, the results are more likely to agree in moderate to advanced disease. On the contrary, they often disagree in early stages when structural changes are subtle. Thus, OCT results classified as within normal range at initial visits in early stages do not necessarily indicate the absence of glaucomatous structural damage. It may only mean that the magnitude of the changes is still below the threshold of detection by OCT. Monitoring with serial scans over time is then required for OCT to detect an abnormality, when the device reaches its minimum sensitivity threshold. In addition, change within the normal range beyond the change expected from aging may also be an important sign of early disease. Therefore, it is important to develop methods to optimize OCT’s ability to differentiate healthy eyes from eyes with early glaucoma.

**Fig. 1:**
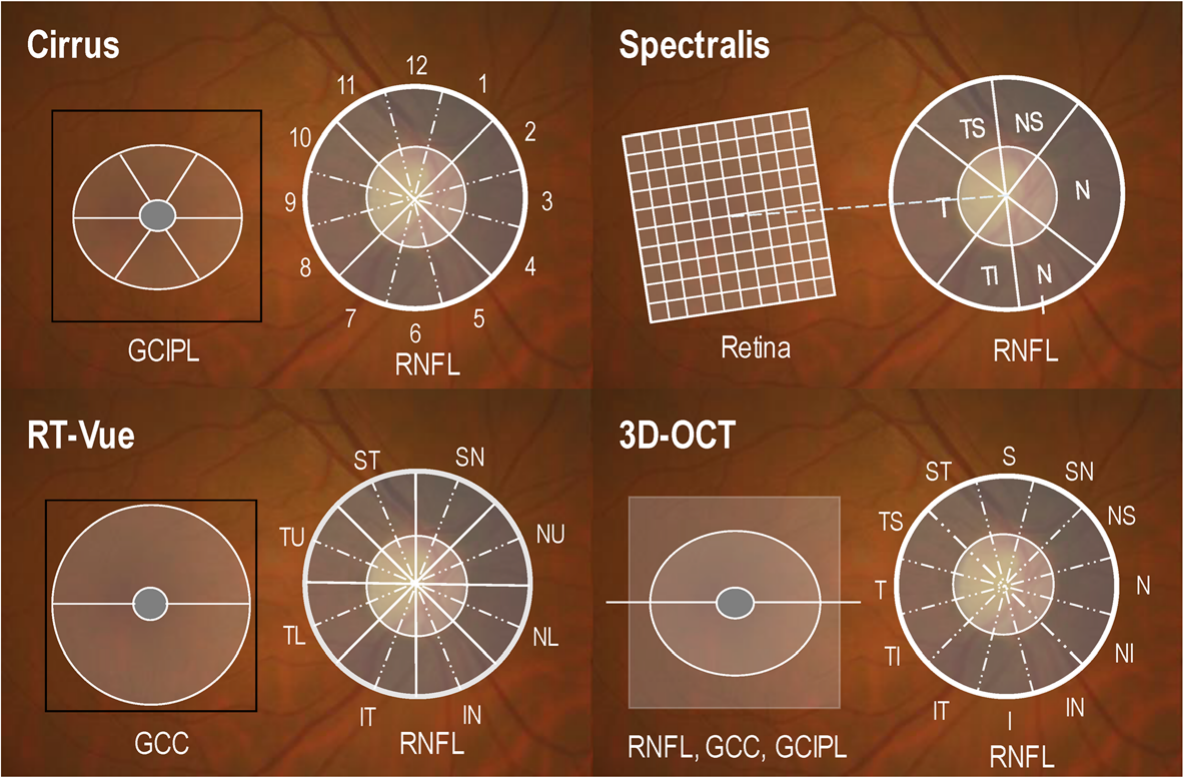
Location of scans and parameters measured by four selected SDOCT devices. Peripapillary scan for measuring RNFL thickness (overall and sectoral) and GCIPL thickness on Cirrus HD-OCT (top let), macular retinal thickness grid on Spectralis (top right), GCC on RTVue (bottom left), and macular RNFL, GCC and GCIPL on Topcon 3D-OCT (bottom right). The same scan centered on the ONH is also used to quantify ONH parameters

The availability of refined statistical methods allows the development of combinatorial algorithms as tools for disease risk categorization, diagnostic classification, and prognostic determination. These methods combine information from single parameters to enhance diagnostic accuracy. Although there is still a shortage of data, available evidence shows that combining individual SDOCT parameters using various methods can offer improved diagnostic performance for early glaucoma. Such an approach minimizes the clinician’s challenge of mentally integrating and processing the panoply of clinical information and OCT data from various parameters when attempting to determine whether a subject has glaucoma or not. This challenge is expected to be greater should OCT glaucoma modules include additional parameters in the future. The sections below present available data on detection of early glaucoma using a combination of SDOCT parameters. Figure 1 shows locations of the scans on four selected SDOCT platforms and the anatomical structure from which the parameters are measured.

## AND- and OR-logic combinations

AND- and OR-Logic are binary concepts and basic operations of Boolean algebra. In this framework *x* AND *y* = 1 if both *x* = 1 and *y*= 1, so *x* AND *y*= 0 if *x*, *y*, or both = 0. *x* OR *y*=1 if *x* = 1 and y = 0 or the opposite, or if both *x* and *y*=1; so *x* OR *y*= 0 if both *x* and *y*=0. AND- and OR-Logic combination methods have been investigated as means to improve the diagnostic discriminating ability of SDOCT parameters. Mwanza et al. used this approach to assess how GCIPL parameters performed in discriminating between 50 patients with early perimetric glaucoma and 49 age-matched normal subjects when used individually or in combination with peripapillary RNFL or ONH parameters measured with Cirrus HD-OCT (Carl Zeiss Meditec, Inc., Dublin, California, USA) [23]. The results indicated that pairing the minimum GCIPL and average RNFL, the minimum GCIPL and rim area, or the minimum GCIPL and inferior quadrant RNFL through OR-Logic method improved the sensitivity, negative predictive value (NPV) and negative likelihood ratio (NLR) relative to the best single GCIPL, RNFL, and ONH parameters, without significantly affecting the specificity. The binary OR-Logic combination of minimum GCIPL and average peripapillary RNFL provided the best overall sensitivity (94%), specificity (85.7%), positive likelihood ratio (PLR, 6.58), and NLR (0.07) compared to the best single GCIPL (minimum: 82%, 87.8%, 6.69, and 0.21), RNFL (inferior quadrant: 74%, 95.9%, 18.1, and 0.27), ONH (rim area: 68%, 98%, 33.3, and 0.33), and best AND-Logic combination (minimum GCIPL + inferior quadrant RNFL: 64%, 100%, infinity, and 0.36). The same approach was used by Jeoung et al., who reported that combining average RNFL and minimum GCIPL measured with Cirrus HD-OCT achieved significantly higher sensitivity (81.1%) and specificity (97.5%) than other OR-Logic and AND-Logic combinations, and single parameters [24]. The findings by both Mwanza et al., [23] and Jeoung et al., [24] suggest that AND-Logic combinations are associated with low diagnostic performances in early glaucoma, likely because of the disagreement between RNFL and GCIPL results at this stage of the disease. From the practical standpoint, the findings also suggest that the diagnosis of early glaucoma should be considered in the presence of either abnormal GCIPL or RNFL parameters, not necessarily both combined.

**Fig. 2:**
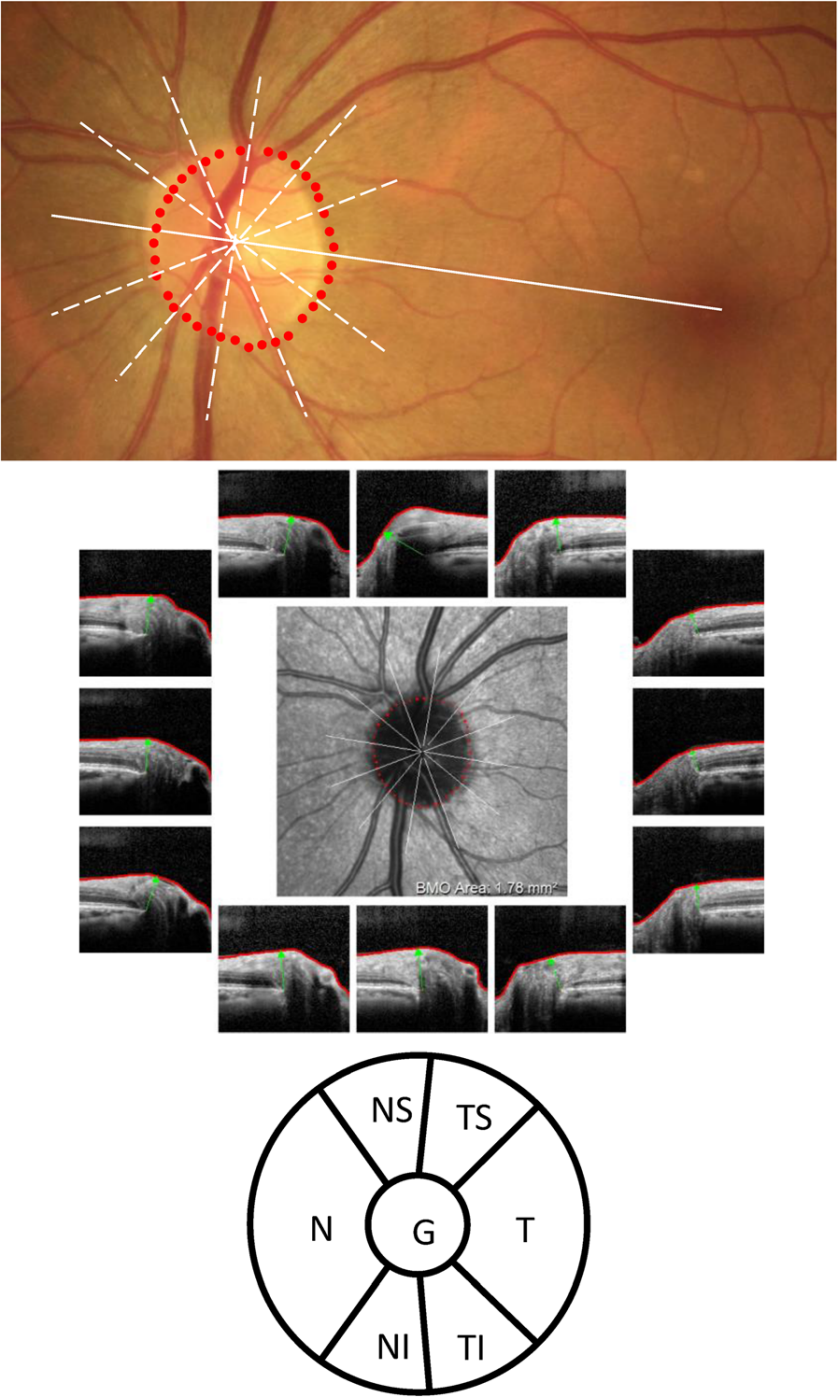
Quantification of minimum rim width (MRW) with Spectralis OCT. OCT fundus photograph (top panel) with disc margin (red dots) as the device will place it. MRW analysis with B-scans corresponding to the 12 clock-hours where the red line represents the internal limiting membrane (LM), the green arrow represents the MRW extending from the Bruch’s membrane opening (BMO) to the ILM (middle panel). The bottom panel shows the sectors for which MRW is generated by the device (same sectors as peripapillary RNFL thickness). Image courtesy of Alexandre Reis, MD, Department of Ophthalmology, University of Campinas, Campinas, Brazil

The MDB is a recently described SDOCT three-dimensional (3D) quantitative neuroretinal rim parameter, although it was first mentioned a decade ago [25, 26]. It is captured with high-density raster scan (i.e., 193 raster line volume scan) with Spectralis OCT (Heidelberg Engineering GmbH, Heidelberg, Germany) and represents the shortest distance between the internal limiting membrane (ILM) and the Bruch’s membrane/retinal pigmented epithelium (BM/RPE) termination [16]. It differs from the MRW, a 3D neuroretinal rim parameter obtained with a low-density ONH scan made of 24 radial lines, defined as the shortest distance between the ILM and the BMO [27]. The MRW uses the BMO to determine the disc margin whereas the MDB uses the RPE/BM complex as disc margin [16, 25]. Although by itself it distinguishes normal eyes from eyes with early glaucoma well (area under the curve of the receiver operating characteristics or AUC of 0.952 and sensitivity of 77.4% at 95% specificity for global MDB thickness), AND-Logic combinations of MDB of the inferior, superotemporal, and superonasal sectors, with the inferior quadrant RNFL performed significantly better (AUC: 0.984) than the best combination of RNFL parameters (0.966) and all single RNFL parameters [28]. The model suggested by Gmeiner and colleagues was created by combining each of the 7 Spectralis BMO-MRW parameters (global, temporal superior, nasal superior, nasal, nasal inferior, temporal inferior, and temporal) (Fig. 2) to its corresponding RNFL parameter [29], based on the following formula:

$$ BMOMRW+ RNFL\kern0.34em Thickness\times \kern0.28em \left( Mean\kern0.34em BMOMRW\kern0.34em Control/ Mean\kern0.34em RNFL\kern0.34em Thickness\kern0.34em Control\right){\displaystyle \begin{array}{c}\\ {}\end{array}} $$

The combined parameters were compared to single ones for their ability to distinguish healthy subjects and patients with pre-perimetric glaucoma. The overall best multivariable parameter resulted from the combination of global parameters (AUC: 0.849, sensitivity at 90% and 95% specificity: 56% and 42%), which increased the performance, but non-significantly, compared to global BMO-MRW (0.821, 52% and 28%) and global RNFL (0.839, 50% and 44%). This method is, in fact, an AND-Logic strategy, although the combinations are limited to BMO-based parameters of the same location.

## Machine learning classifiers and linear discriminant analysis

Imaging data are commonly used in medical decision-making for both diagnosis and treatment and monitoring of diseases. Machine learning classifiers (MLCs) (i.e. linear regression, logistic regression, decision trees, Random Forest, support vector machines, artificial neural networks) and linear discriminant analysis (LDA) are well-established analytical methods for combining input parameters into discriminant functions for classification of patients into groups. Fang et al., studied 34 eyes with early glaucoma and 42 normal eyes and assessed the discriminating abilities of single ONH, RNFL, and GCC parameters measured with RTVue OCT (Optovue Inc., Fremont, California, USA) [30]. Vertical cup-to-disc ratio (VCDR) (AUC: 0.930 and 79.4% sensitivity at 95%), average RNFL (0.915 and 76.5%) and rim area (0.913 and 61.8%) were the best single discriminants. Their combination using a logistic regression model improved the discriminating ability (0.949 and 82.4%) relative to the best single parameter, but the increase was not statistically significant. The disadvantage of this approach is that the choice of parameters used in the combination ignores other factors that may also contribute to the improvement of the diagnostic performance. A recent study compared the diagnostic performance of 19 individual ONH and RNFL Cirrus OCT parameters and a multivariable predictive model using logistic regression with backward elimination technique in a study population of African Americans (103 healthy and 52 with early glaucoma) [31]. The best combination included age, disc area, and RNFL parameters and the multivariable model was defined as:

$$ 0.147+0.73 SQ\kern0.24em RNFL+0.002 CH8\kern0.56em RNFL+0.016 CH12\kern0.56em RNFL+0.045 CH1\kern0.56em RNFL+0.001 CH6\kern0.56em RNFL+2.409 Disc\kern0.34em Area+0.098 Age $$

where SQ is superior quadrant and CH is clock-hour. Despite the multivariable model having an improved performance (AUC: 0.892) compared to the best single RNFL parameters (clock-hour 12: 0.868; inferior quadrant RNFL: 0.857; and average RNFL: 0.855), the improvement was not statistically significant. Individual GCIPL parameters were not included in the logistic regression analysis. It is unclear whether adding inferotemporal GCIPL (AUC: 0.936) would have further improved the performance of the combination. In another investigation, the diagnostic performances of linear discriminant analysis (LDA) and Classification And Regression Tree (CART) were compared to those of single Cirrus HD-OCT ONH and RNFL parameters in early glaucoma [32]. Both the CART model (0.99) and the LDA (0.94) discriminated better than any of the single parameters (AUCs: 0.61–0.89). They also had much lower misclassification rates than single parameters. The CART model included thicknesses of the average, superior, inferior, and nasal quadrant RNFL, disc area, VCDR, cup volume, and RNFL symmetry. LDA combined disc area, rim area, average CDR, VCDR, inferior quadrant RNFL, superior quadrant RNFL, and average RNFL in the following equation:

$$ {\displaystyle \begin{array}{c}1.56 Disc\kern0.34em Area-1.83 Rim\kern0.28em Area-6.21 Average\kern0.28em CDR+5.12 VCDR\\ {}-0.022 SQ\kern0.28em RNFL\kern0.34em Thickness-0.031 IQ\kern0.28em RNFL\kern0.34em Thickness\\ {}+0.016 Average\kern0.34em RNFL\kern0.34em Thickness\end{array}} $$

ONH, peripapillary RNFL, and GCC parameters measured with RTVue were also assessed by Huang et al. for their ability to differentiate normal from glaucomatous eyes, as single parameters and after their combination using LDA [33]. Although glaucomatous eyes were classified as stage 1 (MD: − 5 to − 0.01 dB) and stage 2 (MD: − 12 to − 5.01 dB) on the Bascom Palmer Modified Glaucoma Staging System [34], the MD of the group was − 3.30 ± 2.64 dB, indicating that they all had early glaucoma[Hodapp, 1993 #464]. Their final linear discriminant function was as follows:

$$ -4.332-0.969 Disc\ Area+0.17 ST1\  RNFL+0.22 ST2\  RNFL+0.01 NU2\  RNFL+0.012 IT1\  RNFL+0.048 Standard\ Deviation\ of\ Superior- Inferior\ Hemisphere\  GCC $$

This combination provided an overall better diagnostic performance (AUC: 0.970, sensitivity: 86.3%, and specificity: 95.9%) in early glaucoma than the best single variables (0. 919, 81.5%, and 87.8% for average RNFL; 0.871, 75.3% and 90.5% for inferior hemisphere GCC; 0.854, 71.9%, and 91.9% for VCDR). Yoshida et al. also used the random forests classification method to investigate the discrimination between 126 glaucomatous and 84 normal eye using a total of 151 peripapillary RNFL, macular RNFL, and GCIPL parameters measured with 3D-OCT 1000 (Topcon Corp., Tokyo, Japan) [35]. The method determined that 81 of the 151 parameters (average RNFL; mean, superior and inferior hemiretina macular RNFL; mean, superior and inferior and hemiretina GCIPL; grid macular RNFL in the inferior and superior temporal areas; grid GCIPL in the inferior and superior temporal areas; superior, nasal and inferior quadrant peripapillary RNFL; 30^o^ superotemporal, superonasal, nasal superior, inferior and inferotemporal peripapillary RNFL) were significant predictors of early glaucoma. The diagnostic performance of the random forests combination (AUC: 0.985, sensitivity: 92.9%, specificity: 96.0%) was significantly larger than that of macular RNFL (AUC: 0.934). While random forests are considered an effective MLC algorithm with higher classification accuracy, its prediction performance beyond the limits of the response values in the training data is weak, particularly when used for regression tasks. Overall, it appears from these studies that combination of single OCT parameters using MLCs and LDA allows incremental diagnostic performance in early glaucoma. The magnitude of the improvement varies from one method to another based on the type of device used; the original parameters entered in the model, and the characteristics of the population. Table 1 summarizes the main features of selected combinatorial models discussed below.

**Table 1:** Summary of main features of models combining OCT parameters for the diagnosis of early glaucoma

Model	Analytical Method	Proposed Combination	Predicted Probability Cutoff Points	AUC 95% CI width	Strength	Weakness	Validation
GSDI [37]	Multivariable logistic regression	Average RNFL+GCC; focal loss volume RNFL+GCC; VCDR	Not provided	–	Improved diagnostic ability	Inter-variable collinearity	Internal
OCT Glaucoma Diagnostic Calculator [39]	Multivariable logistic regression	Age, color code for SN, ST, and min GCIPL, CDR, and values of CDR, VCDR, IT GCIPL and inferior quadrant RNFL.	< 0.3 = low; 0.3–0.6 = intermediate; > 0.6 = high	0.046	Improved diagnostic ability	Inter-variable collinearity	Internal
UNC OCT Index [36]	Exploratory Factor Analysis; multivariable logistic regression	Composite RNFL (average, superior and inferior quadrants), composite ONH (VCDR, CDR, rim area), composite GCIPL (all 8 parameters), age	≤0.34 = low; > 0.34 = high	0.011	Minimized inter-variable collinearity, improved diagnostics ability	Some information in the original set of variables may be lost when running the factor analysis	Internal, external
Baskaran et al. [32]	Classification And Regression Tree	RNFL (superior, inferior and nasal quadrant, symmetry); ONH (disc area, VCDR, cup volume)	Not provided	0.01	Low misclassification rate; improved diagnostic ability	Inter-variable collinearity	Internal
Baskaran et al. [32]	Linear Discriminant Analysis	RNFL (average, IQ, SQ); ONH (VCDR, CDR, disc and rim area)	Not provided	0.02	Low misclassification rate; improved diagnostic ability	Inter-variable collinearity	Internal
Blumberg et al. [31]	Logistic regression	Disc area; RNFL (superior quadrant, clock-hours 8, 12, 1, 6); age	Not provided	0.247	Improved diagnostic ability	Wide AUC 95% CI	Internal
Fang et al. [30]	Logistic regression	Average RNFL, VCDR, rim area	Not provided	0.109	–	Variables chosen arbitrary. Wide AUC 95% CI. Inter-variable collinearity.	No
Huang et al. [33]	Linear Discriminant Analysis	RNFL (ST1 and 2, NU2, IT1), disc area, standard deviation of superior and inferior hemispheric GCC	≤0.131	0.045	Improved diagnostic ability	Inter-variable collinearity	No
Yoshida et al. [35]	Random Forest classification	ONH, RNFL, and GCIPL	Not provided	0.028	Improved diagnostic ability	Risk of overfitting	Internal

*AUC* = area under the curve; *CI* = confidence intervals; *GSDI* = Glaucoma Structural Diagnostic Index; *RNFL* = retinal nerve fiber layer; *GCC* = ganglion cell complex; *VCDR* = vertical cup-to-disc ratio; *GCIPL* = ganglion cell-inner plexiform layer; *CDR* = cup-to-disc ratio; *UNC* = University of North Carolina

## The UNC OCT index

**Fig. 3:**
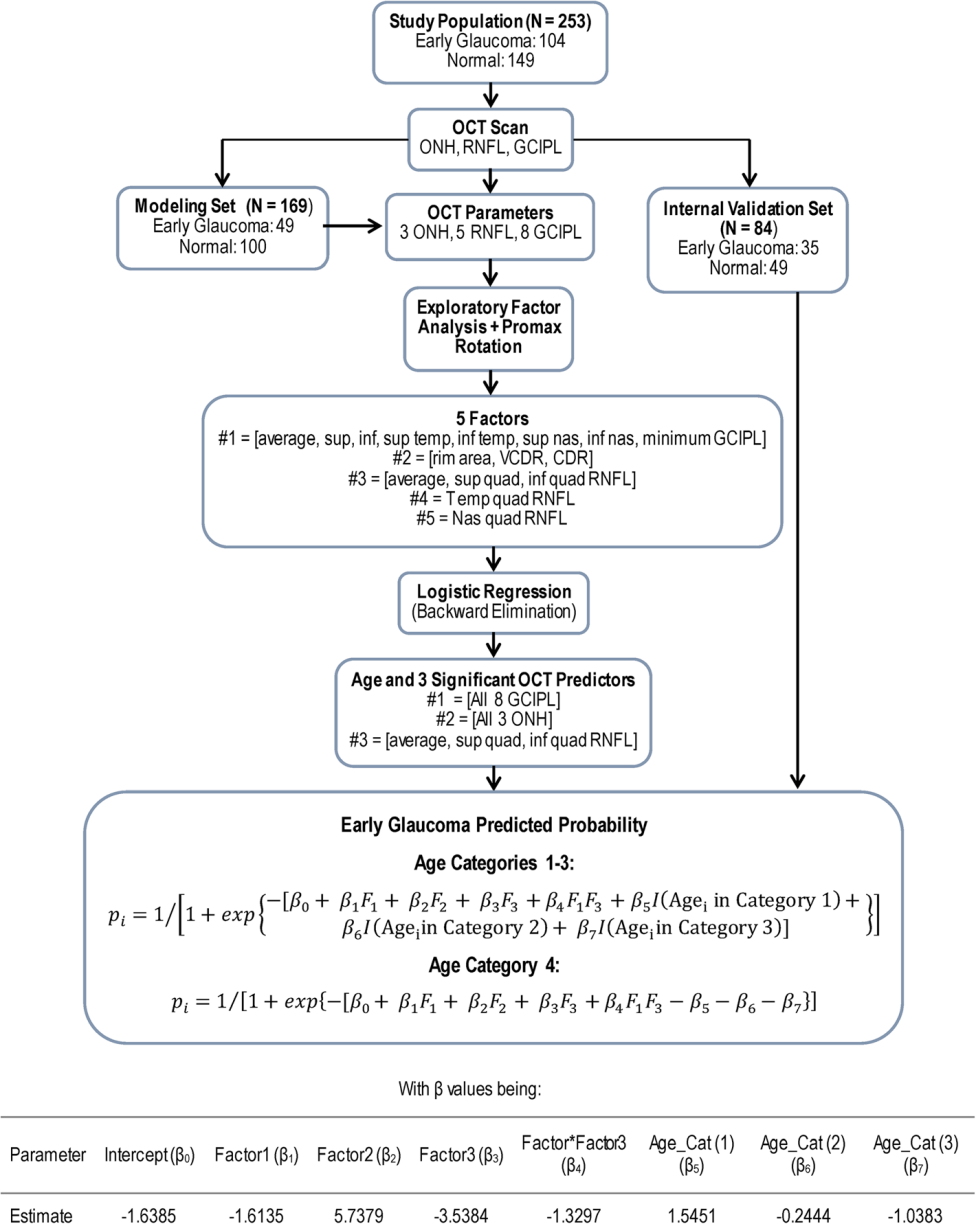
Diagram illustrating the steps of the UNC OCT Index algorithm including the OCT parameters used, the modeling analytical methods (exploratory factor analysis with promax rotation, logistic regression with backward elimination technique the final formula for deriving the predicted probability and internal validation) and final multivariable model for deriving the predicted probability

**Fig. 4:**
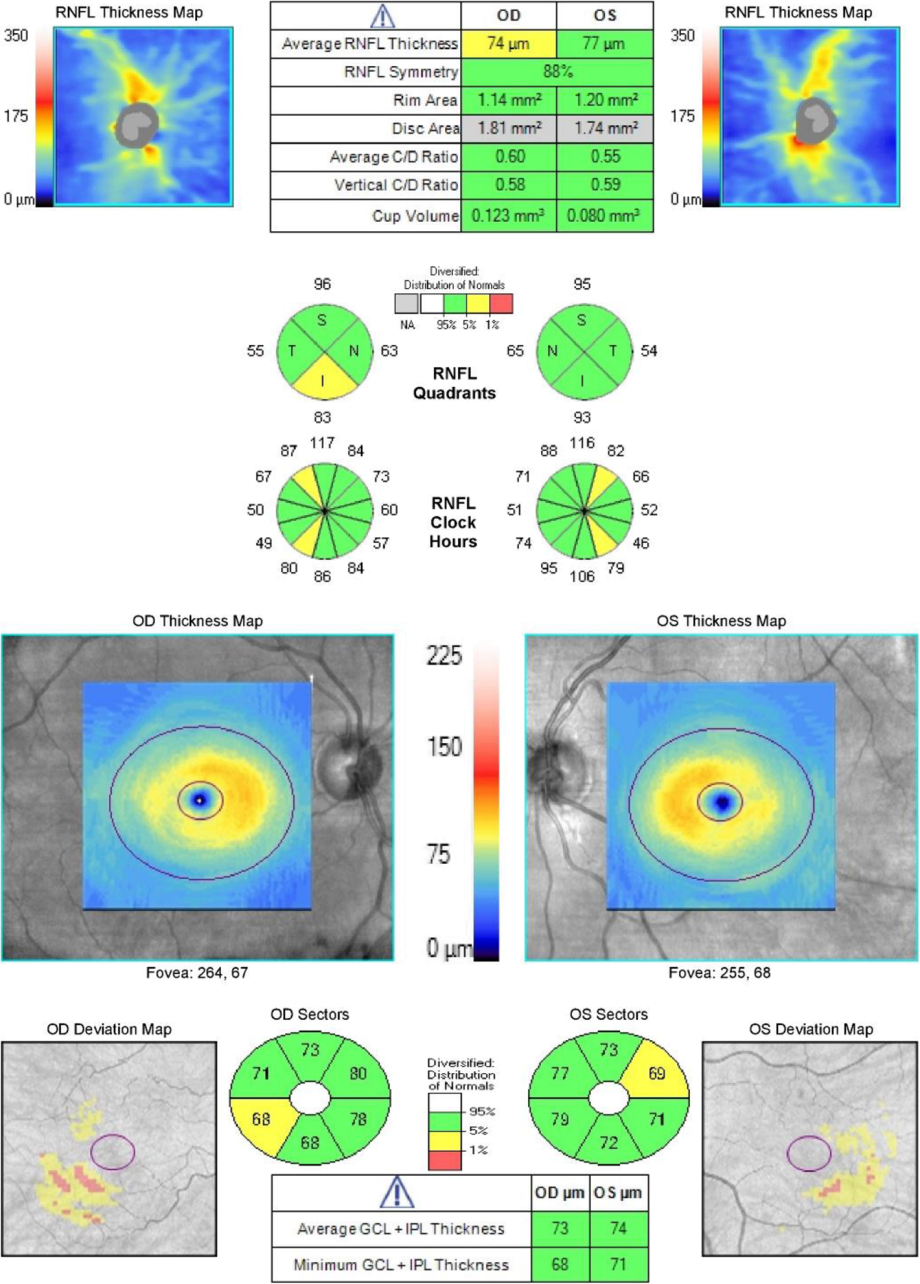
Cirrus OCT report of a 70-year old patient suspected of having glaucoma in both eyes. Visual fields are normal (MD: 0.56 dB in OD and − 0.89 dB in OS). In OD the average, superior quadrant and clock-hours 11 and 7 RNFL and inferotemporal GCIPL thicknesses are borderline, ONH topographic measurements are within normal range. In OS, all measurements are within normal range except RNFL thickness in clock-hours 1 and 5 and GCIPL thickness in the superotemporal sector that are borderline. Application of the UNC OCT Index algorithm yielded predicted probabilities of 0.765 (0.339–0.954) for OD and 0.087 (0.014–0.382) for OS, suggesting high likelihood of glaucoma in OD and low such likelihood in OS

The UNC OCT Index is a combinatorial paradigm that was developed to facilitate the diagnosis of early glaucoma [36]. The significant steps for constructing the model is summarized in Fig. 3. Briefly, the model inputs age and 16 SDOCT quantitative parameters (5 peripapillary RNFL, 8 GCIPL, and 3 ONH). Because of high correlation (positive and negative) between these parameters, they were first submitted to exploratory factor analysis (EFA) with promax rotation to extract latent factors accounting for a large proportion of the variability seen in the original set of parameters. This process identified 5 latent factors accounting for 94.1% of the total variability. Fitting a multivariable logistic regression model with these 5 factors as explanatory variables and glaucoma status as the dependent variable (early glaucoma vs. normal status) identified 3 of the elements as significant predictors of early glaucoma. Using the final formula in Fig. 1, the algorithm instantly and automatically outputs a predicted probability for early glaucoma that defines the UNC OCT Index. The index is a continuous value between 0.0 and 1.0, 0 being no probability of glaucoma and 1 being 100% probability of glaucoma. This model has determined 0.34 as the predicted probability cutoff. Values below 0.34 and those above 0.34 suggest low and high likelihood that the observed structural changes are glaucomatous, respectively. The UNC OCT Index differentiated eyes with early glaucoma from normal eyes better than all single parameters both in the modeling and internal validation sets, based on AUC (0.995 vs. 0.943), sensitivity (98.6% vs. 89.9% at 95% specificity), Akaike Information Criterion (AIC, 43.3 vs. 59.6), median 95% prediction interval length (PIL: 0.05 vs. 0.095–0.15). The robustness of the UNC OCT Index has also undergone an independent validation using a separate cohort of normal eyes and two cohorts of glaucomatous eyes with milder visual field deficit (group 1 MD: - 1.3 ± 1.3 dB and group 2 MD: − 0.7 ± 1.0 dB) than eyes used in the modeling group (MD: − 3.19 ± 1.69 dB). The AUC and sensitivity at 95% specificity of the UNC OCT Index were 0.96 and 85.4% in patients with visual field mean deviation ≥ − 4 dB and 0.95 and 81.7% in those with mean deviation > − 2 dB. Relative to the UNC OCT index, the diagnostic performance indices of the best single variables from each anatomic area the two ≥ − 4 dB group were 0.93 (*P* = 0.05) and 0.92 (*P* = 0.06) for VCDR, 0.92 (*P* = 0.014) and 0.91 (*P* = 0.03) for average RNFL, and 0.91 (*P* = 0.009) and 0.90 (*P* = 0.026) for minimum GCIPL. The sensitivities of the best single parameters were all significantly (all *P* ≤ 0.008), except for rim rea (*P* = 0.07). The results of the independent validation confirmed the effectiveness of the UNC OCT Index combinatorial algorithm over that of single OCT parameters in detecting early glaucoma. The algorithm is stable regarding accuracy and computational speed, and allows more OCT and/or non-OCT parameters to be added as necessary. It is a promising way forward for improving the diagnostic performance of OCT information, and it could be a useful tool for clinical decision-making in glaucoma practice. Figure 4 shows Cirrus HD-OCT data obtained in a glaucoma suspect in whom the UNC OCT Index algorithm suggested a high probability that the right eye was likely glaucomatous (predictive probability: 0.768) whereas the left eye was likely non-glaucomatous (predictive probability: 0.087).

## The Glaucoma structural diagnostic index (GSDI)

The GSDI is a tool developed to improve glaucoma diagnostic accuracy using a combination of SDOCT ONH, peripapillary RNFL, and GCC parameters [37]. The parameters were measured with RTVue OCT in glaucomatous eyes (*n* = 236), a reference normal eye population (*n* = 105), and a cohort of normal eyes (*n* = 118). The multivariable logistic model used to construct the GSDI identified the following 3 significant predictors: 1) composite overall RNFL + GCC thickness, 2) composite RNFL focal loss volume (FLV) + GCC FLV, and 3) VCDR. The final model function was defined as:

$$ -0.74 Composite\ Overall\ Thickness+0.70 Composite\  FLV+3.37 VCDR-3.69 $$

The overall diagnostic accuracy of the combination of these parameters (0.922) was significantly better than that of the best single parameter (nerve fiber layer global loss volume, NFL GLV: 0.896). Below stage 2 of the Glaucoma Severity Staging 2 (GSS2) [38], which may be regarded as early glaucoma, the GSDI was at most 0.874 with a sensitivity of 60.7% at 95% specificity although it was not clear how it compared to single variables since their performances at this stage were not provided.

## The OCT Glaucoma diagnostic calculator

The OCT Glaucoma Diagnostic Calculator was proposed as a tool for the detection of glaucoma regardless of the stage of the disease [39]. It is based on a multivariable predictive model that uses a combination of Cirrus HD-OCT ONH, peripapillary RNFL thickness, and macular GCIPL thickness parameters. A total of 17 parameters were evaluated. The development and validation of this model included data of 500 healthy eyes and a separate group of 187 glaucomatous eyes of all severity stages. The study and validation groups covered 92 and 37 stage 1 glaucomatous eyes based on the GSS2, respectively. Three different models were evaluated and compared, with model #1 using quantitative data only, model #2 qualitative data only, and model #3 a combination of qualitative and quantitative information. Model #3 proved to be the best and used a combination of age, color classification code for superonasal GCIPL, superotemporal GCIPL, minimum GCIPL and average CDR; thicknesses of inferotemporal GCIPL and inferior quadrant RNFL; and values of average CDR and VCDR. Colors are based on the classification relative to the normative database and are given scores of 0 for green (all parameters), 1 for yellow (all parameters), 2 for red (all parameters), and 3 for gray (average CDR). Though details were not provided, it was reported that this model significantly outperformed all single parameters in early glaucoma. The predicted probability of the model 3 is given as:

$$ {\displaystyle \begin{array}{c}{e}^{\hat{\mkern6mu}}\Big(0.905+0.044 Age-1.477\left( SNGCC= yellow\right)-1.190\left( SNGCC= red\right)\\ {}+1.403\left( STGCC= yellow\right)+1.095\left( STGCC= red\right)\\ {}+1.455\left( MCGC= yellow\right)+1.109\left( MCGC= red\right)\\ {}+0.006\left( CDAC= yellow\right)+2.231\left( CDAC= red\right)\\ {}+0.583\left( CDAC= gray\right)-0.034 ITGC-0.035 IRNFL\\ {}-0.099 CDA\left(\times 100\right)+0.117 VCD\left(\times 100\right)/\Big(1\\ {}+{e}^{\hat{\mkern6mu}}\Big(0.905+0.044 Age-1.477\left( SNGCC= yellow\right)\\ {}-1.190\left( SNGCC= red\right)+1.403\left( STGCC= yellow\right)\\ {}+1.095\left( STGCC= red\right)+1.455\left( MCGC= yellow\right)\\ {}+1.109\left( MCGC= red\right)+0.006\left( CDAC= yellow\right)\\ {}+2.231\left( CDAC= red\right)+0.583\left( CDAC= gray\right)\\ {}-0.034 ITGC-0.035 IRNFL-0.099 CDA\left(\times 100\right)\\ {}+0.117 VCD\left(\times 100\right)\left)\right)\end{array}} $$

with SNGCC, STGCC, and MCGC being colors of the superonasal, superotemporal, and minimum GCIPL; respectively. CDAC is the color of the average CDR; ITGC, IRNFL are values of the inferotemporal GCIPL and inferior quadrant RNFL thicknesses, respectively. CDA C/D and CVD are values of the average and vertical CDR, respectively. The calculator outputs a probability classification that ranges between 0.00 and 1.00 and labelled the result as positive (high probability of glaucoma), negative (low probability), or inconclusive (intermediate probability). This model achieved an AUC of 0.937 and sensitivity of 77.8% at 95% specificity compared to 0.877 and 59.8% (all *P* < 0.001) for inferotemporal RNFL.

## Conclusions and future perspectives

Multiple SDOCT parameters from various ocular anatomic areas are now available that clinicians use for distinguishing between diseased and non-diseased subjects, particularly in early stages. The challenge for diagnosing early glaucoma clinically and the difficulty of interpreting several parameters that yield conflicting information have been the impetus for investigating various ways to improve diagnosis of early glaucoma while alleviating the clinician’s tasks. A desirable approach has been to combine multiple diagnostic tests or parameters from the same test to obtain an optimal composite diagnostic test with higher sensitivity and specificity that detects the presence of the disease more accurately. This mini-review has outlined how combining information from different structural OCT parameters may be a complementary tool for the diagnosis of early glaucoma. It transpires from this review that: (1) combinatorial models of OCT structural parameters for glaucoma have so far remained research tools, (2) such models for early glaucoma should be prioritized, as the clinical diagnosis of moderate to advanced glaucoma is generally straightforward, and (3) combination of single parameters into composite improves the diagnostic ability of OCT in early glaucoma. The improvement should not be judged based on AUC alone, but together with sensitivity, specificity and other diagnostic performance indices. However as of to date, just as there is no agreed-upon unique standard guideline for diagnosing early glaucoma with the aid of single OCT parameters, there is equally no consensus yet on what constitutes the best combinatorial model for OCT parameters. Although some patients with early glaucoma can be diagnosed with a single baseline visit, many of them will be diagnosed after follow-up and detection of progressive glaucomatous changes to the structures affected by the disease even if they remain in the normal range for age. The question for future research is whether OCT combinatorial models may help detect progression earlier than single parameters in early glaucoma. Despite a few recent reports to the contrary [13, 40–42], it is generally known that glaucomatous structural changes are more difficult to detect in moderate to advanced disease. Thus, future research may also need to investigate whether combinatorial models may improve detection of structural progression in moderate to advanced glaucoma. It is an improvement in detection of early glaucoma and progression throughout the course of the disease that will allow earlier diagnosis and timely initiation or adjustment of treatment, to reduce the burden of glaucoma-related visual loss and its consequences.

## Abbreviations

AIC: Akaike’s Information Criterion
BM: Bruch’s membrane
CART: Classification and regression tree
CDR: Cup-to-disc ratio
EFA: Exploratory factor analysis
FLV: Focal loss volume
GCC: Ganglion cell complex
GCIPL: Ganglion cell-inner plexiform layer
GLV: Global loss volume
GSS: Glaucoma staging system
ILM: Internal limiting membrane
LCCI: Lamina cribrosa curvature index
LCD: Lamina cribrosa depth
LDA: Linear discriminant analysis
MD: Mean deviation
MDB: Minimum distance band
MLC: Machine learning classifier
NFL: Nerve fiber layer
NLR: Negative likelihood ratio
ONH: Optic nerve head
PIL: Prediction interval length
PRL: Positive likelihood ratio
RNFL: Retinal nerve fiber layer
RPE: Retinal pigmented epithelium
SDOCT: Spectral domain optical coherence tomography
UNC: University of North Carolina
